# The componential nature of arithmetical cognition: some important questions

**DOI:** 10.3389/fpsyg.2023.1188271

**Published:** 2023-09-14

**Authors:** Ann Dowker

**Affiliations:** Experimental Psychology, University of Oxford, Oxford, United Kingdom

**Keywords:** arithmetical cognition, components of arithmetic, domain-general abilities, domain-specific abilities, non-symbolic and symbolic quantity representation, arithmetical operations, arithmetic concepts, dyscalculia

## Abstract

Research on typically developing children and adults and people with developmental and acquired dyscalculia converges in indicating that arithmetical ability is not unitary but is made up of many different components. Categories of components include non-symbolic quantity representation and processing; symbolic quantity representation and processing; counting procedures and principles; arithmetic operations; arithmetical knowledge and understanding; multiple forms and applications of conceptual knowledge of arithmetic; and domain-general abilities such as attention, executive functions and working memory. There is much evidence that different components can and often do show considerable functional independence, not only in developmental and acquired dyscalculia, but in typically achieving children and adults. At the same time, it is possible to find complex interactions and bidirectional relationships between the different components, including between domain-specific and apparently domain-general abilities. There is a great deal that still needs to be discovered. In particular, we need to learn more about the origins in infancy of subitizing and approximate magnitude comparison, the extent to which these interact, the extent to which they may be further divisible, and the extent and ways in which they themselves may develop with age and the extent to which they may influence later-developing components. There also needs to be a lot more research on exactly how domain-general and domain-specific abilities contribute to mathematical development, and how they interact with one another.

## Introduction

Research on typically developing children and adults and people with developmental and acquired dyscalculia converges in indicating that arithmetical ability is not unitary but is made up of many different components (Dowker, 2005, 2015). For example, patients with brain disorders can show selective deficits in dealing with arithmetical facts and procedures, while showing unimpaired conceptual knowledge of arithmetic (Cappelletti et al., 2005). They may show impairment just in factual knowledge with unimpaired procedural and conceptual knowledge (Warrington, 1982). They may show preserved factual knowledge with impaired conceptual knowledge (Delazer and Benke, 1997). These are just a few of the discrepancies that can occur in patients. Perhaps more surprisingly, typically developing children and adults can show discrepancies that are almost as great.

This paper will discuss some of the more important components of arithmetical cognition, and some of the ways in which they can dissociate in typical and atypical development and functioning. It is not intended to provide a complete list of these components, from the point of view of psychology, education or mathematics. Indeed it is questionable whether a full and definitive list of such components can exist.

The main components of arithmetical cognition are sometimes grouped into the categories of factual, procedural and conceptual knowledge (Delazer, 2003) but each of these categories may be further divided into numerous other categories. Thus, factual knowledge may include memory for different types of arithmetical facts, such as addition and multiplication; and also the names given to different operations Procedural knowledge includes written, oral and concrete calculation procedures. Conceptual knowledge, includes of the semantics of word problems; the meaning of the place value system; arithmetical principles such as commutativity, associativity and the addition-subtraction inverse principle, and at its foundations, principles that inform and guide counting.

There is indeed overwhelming evidence that arithmetical ability Is not unitary (e.g., Jordan et al., 2009; Cowan et al., 2011; Gifford and Rockliffe, 2012; Pieters et al., 2012; Dowker, 2015). Rather, it consists of multiple components, including counting principles, counting procedures, solving word problems, fact retrieval, calculation procedures, understanding and using arithmetical principles, and numerous others. Although the different components often correlate with one another, it is possible to demonstrate difficulties in almost any one component without corresponding difficulties in other components. It is, however, true that weaknesses in one component can ultimately affect performance in others. This is partly because difficulty with one component may sometimes lead to individuals failing to perceive, integrate and use relationships between different arithmetical processes; and partly because when children experience failure, even if just in very specific tasks, they may come to perceive themselves as “bad at maths” and develop negative attitudes to the whole subject.

## Different components of arithmetic itself: different operations

Although this article focuses mainly on the componential nature of arithmetical cognition in a broad sense, there is evidence that different specific aspects of arithmetic itself can dissociate. Patients can demonstrate specific impairments in different arithmetical operations: addition, subtraction, multiplication or division. Dehaene and Cohen (1997) and McNeil and Warrington (1994) described patients, who were much more impaired in subtraction than in addition. This cannot bee explained simply in terms of subtraction being more difficult than addition, as Dagenbach and McCloskey (1992) described a patient, who was much more impaired in both addition and multiplication than in subtraction. Van Harskamp and Cipolotti (2001) reported three patients who had specific respective impairments in simple addition, simple subtraction and simple multiplication. Cipolotti and de Lacy Costello (1995) described a patient who had a specific impairment in simple division. Venneri and Semenza (2011) reported a patient who had specific difficulty with multiplication, despite preserved competence not only with addition and subtraction, but also with division, which had sometimes been regarded as dependent on multiplication.

Patients also sometimes show deficits in particular aspects of an arithmetical operation, such as carrying in addition or borrowing in subtraction. For example, Carota et al. (2013) report a patient who had no difficulty in solving large-number subtraction, provided that no borrowing was involved; but had considerable difficulty with smaller number problems that did involve borrowing.

Though most studies of specific weaknesses in arithmetical operations and their components have involved neuropsychological patients, such weaknesses also occur in apparently typically developing children and adults. For example, Dowker (2005) described a university student who had an A grade in mathematics A level and a scaled score of 14 on the Arithmetic subtest of the Wechsler Adult Intelligence Scale. She also obtained extremely high scores outstandingly well on all subtests of Hitch’s (1978) Numerical Abilities Tests, except for the test of Subtraction with Borrowing, on which she scored zero.

The focus of this article is on whole number arithmetic; but fractions and decimals, and the use of arithmetical operations in dealing with them, are usually more difficult for people to handle, both conceptually and procedurally (Hecht, 1998; Siegler et al., 2013; Lortie-Forgues et al., 2015).

## Evidence from research in developmental psychology

In particular, studies of both atypical and typical mathematical development in children have indicated that from an early age, arithmetical cognition is already made up of multiple components (Russell and Ginsburg, 1984; Dowker, 2005; Desoete and Grégoire, 2006; Jordan et al., 2009; Cowan et al., 2011; Gifford and Rockliffe, 2012; Kaufmann et al., 2013; Santos et al., 2022). For example, these include counting, calculation, estimation, fact retrieval, arithmetical reasoning and word problem solving. Moreover, individuals may show strong discrepancies, in either direction, between almost any two components. Although different components usually correlate significantly with one another, and some components typically appear to be easier o than others, there does not seem to be a clear hierarchy of abilities.

Dowker (1998, 2005, 2014) studied 291 unselected primary school children between 5 years 2 months and 9 years 10 months and investigated their performance on three different types of addition task: calculation. Arithmetical estimation and derived fact strategy use. The difficulty of the arithmetic problems presented to individual children for the estimation and derived fact strategy tasks was adapted to their assessed calculation performance levels assessed in a pre-test. Nevertheless, children’s addition calculation performance level correlated significantly with both their estimation and their derived fact strategy use. A strong independent relationship between derived fact strategy use and estimation was also found, even after controlling for addition calculation level. Despite these overall correlations, there were individual children, at all ages and achievement levels, who showed marked discrepancies between scores on all possible pairs of arithmetic tasks. Such discrepancies occurred in all directions: it was not the case that some tasks were invariably more difficult than others.

Dowker (2009) gave similar calculation, estimation and derived fact strategy tests to 204 children with arithmetical difficulties, as identified by their schools. These children were compared with 135 unselected children of similar age and school background from the same schools. The unselected children performed very similarly. to those in the above-mentioned studies. Predictably, the children with mathematical difficulties performed worse on all tasks than the unselected children. Less predictably, they showed even greater functional independence between the different tasks than did the unselected children. Among the children with mathematical difficulties, unlike the unselected children, not only were derived fact strategy use and estimation not independently related; they did not even show a significant correlation before other factors were partialled out. Moreover, standardized arithmetic test performance correlated significantly with both derived fact strategy use and estimation in the unselected group, but not in the group with mathematical difficulties. This was not because of floor effects in the group with mathematical difficulties, as they showed considerable variance in their scores on all tasks.

It may be that in typical mathematical development, the components inform and integrate with each other to a greater extent than they do in delayed or atypical mathematical development. In the latter situation, either the integrative process itself may not work as effectively, or it may be impaired by weakness in the individual components. It would be desirable to investigate whether these differences between children with and with and without mathematical difficulties, with regard to the level of association between components, would be replicated in different samples and age-groups. If so, do weaknesses in individual components impede integration, or does integration failure impede progress in the individual components, or both?

An interesting question is whether numeracy is already componential in the preschool years, with regard to counting procedures and concepts. Dowker (2008) found evidence that it is. She gave 80 four-year-old children an object counting task; Wynn (1992) counting-versus-grabbing task as a measure of understanding the cardinal word principle; an order-irrelevance task; a task where they repeatedly had to say, without counting. How many objects there were, after a new object was added (the task started with 5 objects and was repeated up to 12); and a task where they repeatedly had to say, without counting. How many objects there were, after an object was removed (the task started with 10 objects and was repeated down to 1).

Scores on most tasks correlated significantly, but discrepancies in both directions occurred between almost any two tasks. For almost any pair of tasks, there were children who could perform either one of the tasks but not the other. For example, proficiency in counting objects correlated significantly with performance on the cardinal word principle task, but 22% of proficient counters were “grabbers” on the cardinal word task, and 41% of nonproficient counters were “counters” on the cardinal word task.

Dowker and Grimer (in preparation) carried out a further study, investigating relationships between counting proficiency, the order irrelevance principle, repeated subtraction by 1, error detection and arithmetic in the early primary school years, and examined the possibility of differences in performance for biological and non-biological stimuli. The participants were seventy-five children between. 4 years 3 months and 8 years 5 months (mean age: 6 years 3 months). Children improved significantly with age on all numerical abilities. Most numerical abilities correlated with one another. In particular, counting proficiency correlated significantly with order-irrelevance, error detection, and arithmetic. Order irrelevance also correlated significantly with repeated subtraction by one. However, once again, discrepancies in both directions could be found, within individuals, between almost any two components of numerical ability. Children performed better on the error detection task for biological than non-biological stimuli; other tasks were not affected by this distinction.

## Evidence from neuropsychology for the componential nature of arithmetical cognition: studies of patients

As indicated at the beginning of this article, some strong evidence for the componential nature of arithmetical cognition comes from selective impairments of specific aspects of arithmetical cognition in patients with brain damage. Patients have demonstrated double dissociations between factual and procedural knowledge (Warrington, 1982; Dagenbach and McCloskey, 1992; Van Harskamp and Cipolotti, 2001) and between factual/procedural and conceptual knowledge (Warrington, 1982; Hittmair-Delazer et al., 1994, 1995; Delazer and Benke, 1997; Pesenti et al., 2000; Delazer, 2003; Julien et al., 2010). For example, Puvanendran et al. (2015) describe a patient with poor verbal working memory associated with Broca’s aphasia, who performed very poorly on fact retrieval tasks, but showed excellent use of derived fact strategies.

Dissociations between different components of arithmetical knowledge and processing have been found not only in patients with focal brain lesions but in those with neurodegenerative disorders. Kaufmann et al. (2001) studied people in the early stages of Alzheimer’s disease and found that they showed a variety of dissociations between arithmetic facts, arithmetic procedures, and the understanding of relative magnitudes of numbers. No component was found to be a prerequisite for other components. Papagno et al. (2013) reported a patient with semantic dementia, who was severely impaired in arithmetic fact knowledge but showed preserved quantitative number knowledge as well as outstanding performance in numerical Sudoku. It is probably relevant that this patient showed substantial sparing of the parietal lobes despite severe atrophy of the temporal and frontal regions.

Warrington (1982) studied a patient who had suffered a stroke affecting the left posterior parieto-occipital region of the brain. This patient, “DRC,” had a selective impairment in number fact retrieval, and resulting in difficulties with several aspects of calculation. He performed poorly on Hitch (1978) Test 1, involving whole-number arithmetic. However, he performed similarly to controls on Test 2, involving fraction and decimal arithmetic and Test 3, involving numerical reasoning and numerical magnitude appreciation. He was also unimpaired in arithmetical estimation tasks, Somewhat similarly, Puvanendran et al. (2015) report a patient with Broca’s aphasia resulting in poor verbal working memory, who demonstrated very poor fact retrieval, but excellent derived fact strategy use.

Dehaene and Cohen (1995) studied a patient “Mr. N” with a left subcortical lesion, who had much more extreme problems with calculation than the patients described above. Patient. He could not carry out any exact calculations reliably, even involving very small numbers, such as adding 2 + 2. He could, however, deal accurately with approximations, for example, locating numbers approximately on a number line; comparing the value of two 2-digit numbers; and rejecting wildly inaccurate answers to addition problems. By contrast, another patient, with a left inferior parietal lesion, failed at such approximation tasks, but could still carry out some exact calculations through preserved rote knowledge of some arithmetic facts.

Cappelletti et al. (2012) gave a comprehensive battery of numerical and calculation tasks to 36 were patients with either neurodegenerative disorders or focal brain lesions and to forty healthy controls. All patients, including those with parietal lesions, had intact processing of number quantity. Impaired calculation skills were found in most patients with specific brain lesions, even when these did not involve the parietal lobes. However, most patients with dementia did not show impaired calculation.

People with hippocampal damage, either due to injury or to atrophy (the latter often associated with early Alzheimer’s disease) are often unimpaired in retrieval of well-learned arithmetical facts, such as multiplication tables. There may be a distinction, with regard to arithmetical facts as with regard to other types of factual information, between the ability to lay down arithmetical facts and the ability to retrieve well-consolidated arithmetical facts. Delazer et al. (2019) found that13 out of 16 patients with hippocampal atrophy associated with Alzheimer’s disease showed intact retrieval of consolidated arithmetic facts from memory.

While there is no invariable one-to-one relationship between specific brain areas and specific aspects of numerical cognition, much research suggests the importance of parietal areas, and especially the intraparietal sulcus (IPS) in basic numerical tasks including quantity and numerical comparisons and single digit arithmetic. It may also be important in more complex computational estimation. Ganor-Stern et al. (2020) compared a patient with damage to the left IPS with age-matched controls in a computational estimation task involving estimating whether the results of multi-digit multiplication problems were smaller or larger than given reference numbers. The patient did not show the same distance and size effects as the controls. Moreover, most control participants used both the approximate calculation strategies involving rounding and calculation procedures, and an intuitive approximated magnitude representation of the results. The patient used only the approximate calculation strategies.

## Evidence from neuroscience for the componential nature of arithmetical cognition: brain imaging studies

Brain imaging studies of healthy people also suggest that different brain networks may support different aspects of arithmetic and number processing. Dehaene and colleagues carried out functional MRI and ERP studies of approximation and exact calculation in healthy individuals (Dehaene et al., 1999; Cohen et al., 2000; Stanescu-Cosson et al., 2000). Parietal and frontal areas were more active during approximation than exact calculation. By contrast a distributed set of areas, including a left anterior inferior frontal region and the bilateral angular gyri, showed greater activation during exact calculation. Grabner et al. (2007) carried out a functional MRI study of 28 adults doing mental arithmetic and found that the angular gyrus was most active for problems which they reported solving by retrieval, while a wider frontal–parietal network was activated by problems for which they reported using a calculation procedure.

Liu et al. (2019) carried out an fMRI study, which included an investigation of functional connectivity, and compared the brain areas involved in computation and in processing arithmetical principles, as well as in language processing Arithmetical principles were associated with stronger activation in the left middle temporal lobe and left orbital part of the inferior frontal gyrus than was the case for computation. Computation was associated with greater activation in the bilateral horizontal intraparietal sulcus than was the case for arithmetic principles or language processing. Left temporal–parietal connectivity was stronger for arithmetic principles than computation, while parietal-occipital connectivities were stronger for computation than arithmetic principles.

Amalric and Dehaene (2016) carried out fMRI studies with professional mathematicians, who were asked judge the truth value of mathematical and nonmathematical spoken statements. The bilateral intraparietal sulci and inferior temporal regions were consistently activated by sentences with mathematical content. The level of activation of classical language areas was related to syntactic complexity and not to mathematical content. This suggests that there is a distinct, non-linguistic cortical network associated with mathematical knowledge; though it is, of course, possible that non-mathematicians would have given different results.

Of course, activation of particular brain areas during a particular mathematical task does not necessarily mean that these areas are specialized for particular components of mathematics. They could be supporting non-numerical, domain-general processes relevant to the task in question. Gruber et al. (2001) used functional MRI to compare the areas of the brain that are active in calculation and in non-arithmetical tasks that involved language, visual–spatial processing, attention or memory. They concluded, on the basis of similarities between areas activated by arithmetical and non-arithmetical tasks, that most of the cortical areas that subserve arithmetic “do not exclusively represent modules for calculation but support more general cognitive operations that are instrumental but not specific to calculation.” However: the left dorsal angular gyrus and the medial parietal cortices were more specifically activated by arithmetical tasks.

## Early non-symbolic number processing abilities: foundations for other numerical abilities?

In order to understand the componential nature of numerical ability, it is important to gain a greater understanding of its earliest foundations. We have evidence from developmental studies, discussed above, that numerical abilities are already componential in the preschool years. But are they so from the beginning? One unresolved question is whether symbolic number representation grows directly out of non-symbolic number representation or should be regarded as a separate skill or combination of skills. There is also the perhaps even more fundamental question of whether the primary foundational ability is subitizing or approximate number representation or whether, as seems most likely, both are crucial: i.e., numerical ability is componential from its foundations. Despite the likelihood that both subitizing and the approximate number system are at the foundations of arithmetical development, there is evidence that later arithmetical abilities can show considerable functional independence from both.

Humans’ ability to enumerate visual stimuli involves not only verbal counting, but on two nonverbal quantity recognition processes: the Approximate Number System (ANS) (Dehaene, 1997), which involves the approximate estimation of quantities above 3 or 4 items, and subitizing, which involves the exact recognition of small quantities up to about 3 items. A key question, if we are to understand the nature and foundations of, the componential nature of arithmetical ability, is that of how subitizing and the approximate number system are related to one another in early and later development, and the extent to which later numerical abilities depend on them. When does numerical ability become componential? Do infants start out with a unitary foundation, which eventually diverges, or are the preverbal foundations of early numerical ability componential from the beginning? At present, the evidence, both from studies of infants and young children, and from studies of development and acquired dyscalculia, suggests that both subitizing and approximation are present from the beginning, and that both are important foundations for later arithmetical development. That would suggest that numerical abilities are componential not only in later development but at their foundation.

## Subitizing: is it a foundation?

For example, some studies of both children and adults suggest that non-symbolic number comparison abilities are only weakly related to symbolic comparison and to arithmetic. Lyons et al. (2012) speak of “symbolic estrangement”: an increasing dissociation between symbolic and non-symbolic comparison abilities. Some studies of patients with brain damage suggest that there is no strong relationship between acquired deficits in subitizing and in arithmetic (Cappelletti et al., 2012; Gosling et al., 2023). This section will discuss the degree and ways in which subitizing, and the approximate number system may be necessary to the development of other abilities; the limits to such relationships; and the reasons for these limits.

There are still some disagreements about the nature of subitizing and about how far the subitizing range extends. Kaufman et al. (1949), suggested that it extended up to 6 items. Most more recent researchers have suggested a smaller range, of 3 items (Mandler and Shebo, 1982) or 4 items (Trick and Pylyshyn, 1994). Within the subitizing range, adding an item only increases response time slightly, by between 50 ms and 80 ms (e.g., Mandler and Shebo, 1982). Beyond the subitizing range, adults typically adopt a counting strategy. Resulting in a near linear increase in response time with each item added to the visual scene. Studies have suggested that, after 4 items, response time increases by 250-350 ms per item (Chi and Klahr, 1975; Trick and Pylyshyn, 1994; Wender and Rothkegel, 2000).

Many studies of subitizing have involved infants. Most of these studies indicate that babies can distinguish quantities up to 3 (Strauss and Curtis, 1981; Starkey et al., 1990; Starkey and Cooper, 1995), though there are debates about the level of abstractness of babies’ quantity representations, and the extent to which they represent discrete quantities independently of the total area occupied (Clearfield and Mix, 1999). Even many non-human animal species appear to have some ability to recognize quantities up to 3Adults, and may have a slightly higher subitizing range than infants, either due to their greater experience. or because they are able to label quantities verbally, or both. Starkey and Cooper (1995) proposed a subitizing range of 3 in infants and young children and 5 in adults.

Many researchers consider that different underlying processes support subitizing and counting. Some researchers, such as Balakrishnan and Ashby (1991), have presented an opposite view. Subitizing is a result of rapid parallel processing whereas counting is a much slower serial process. There are several different models of enumeration, which involve different processes for counting and subitizing. For example, Trick and Pylyshyn (1994) proposed the FINST (Fingers of Instantiation) model of enumeration. Parallel indexing of items occurs using four FINST. Enumeration of up to 4 items is therefore fast but after this, the process becomes much slower. The FINSTs must then be serially reallocated to different spatial locations. This reflects a counting strategy being adopted and increased response times. In contrast, Logan and Zbrodoff (2003) believe that subitizing is purely pattern recognition and requires little processing. This debate remains unresolved. Some researchers, such as Clements (1999) have argued that subitizing as a perceptual process may lead to the development of “conceptual subitizing” where smaller readily perceivable quantities are strategically combined and grouped to facilitate the estimation of larger quantities.

Some neuropsychological evidence supports a dissociation between subitizing and counting. Demeyere et al. (2010, 2012) found that damage to the left posterior occipital cortex, bilateral lateral occipital and right superior frontal cortices resulted in deficits subitizing range while damage to the left intraparietal sulcus resulted in deficits in counting. This suggests that at least to some extent, subitizing and counting involve different processes and neural areas.

Evidence from brain imaging studies of healthy individuals is somewhat conflicting. Piazza et al. (2002) found that both subitizing and counting activate a common network involving both occipital and parietal areas. By contrast, Sathian et al. (1999) found that subitizing was associated with activation mainly in occipital areas, while counting was associated with activation mainly in frontal and parietal areas. Gosling et al. (2023) tested eleven healthy control participants and nine chronic patients with acquired brain injury on tasks focused on visual enumeration, addition and multiplication to investigate whether there were relationships between subitizing ability and calculation performance. They found no significant overall correlation between subitizing and either addition or multiplication speed. Two patients did show, a very clear subitizing impairment. One of them showed significant impairments in addition skills, while the other did not. Thus, it appears that, at least in adults who have already developed arithmetical skills, such skills are not invariably dependent on subitizing.

Despite the findings mentioned above, it is likely that the two processes are still linked to some degree. There is substantial evidence that counting relies on the accurate development of subitizing. This may be due to some underlying neuropsychological similarities or the foundational nature of abilities that support numerical representations, one of which is an innate capacity to represent small numerosities. This capacity is demonstrated in subitizing tasks; and Butterworth (2000) proposed that such subitizing ability lies at the foundation of mathematical ability. There are debates about whether subitizing or the ANS appears earliest in infancy and is most crucial to numerical development (see Dehaene, 1997). It is, however, now generally considered that both are present at or near the beginning, and are applied to different sizes of quantities: subitizing to smaller quantities and the ANS to larger quantities.

A number of studies have shown links between subitizing and various other aspects of mathematical ability (Butterworth, 2000, 2005; Moeller et al., 2009; Butterworth et al., 2011; Wilkins et al., 2022). For example, Reeve and Reynolds (2004) found 6% of a randomly selected group of children in their first year of formal schooling did not demonstrate the ability to subitize. When tested 1 year later and 2 years later, those who had not shown evidence of subitizing in infancy were much slower than the others at reading three-digit numbers. Desoete and Grégoire (2006) found subitizing ability at the end of kindergarten to be predictive of mathematical performance in first grade. Hannula Sormunen et al. (2015) found that preschool children’s subitizing predicted their mathematical performance 7 years later. Few studies have examined individual differences in subitizing in typical adults, but Sowinski (2013) did so and found that subitizing correlated with arithmetic fluency.

In line with these findings, problems in subitizing have been identified in some children and adults with developmental dyscalculia. Koontz and Berch (1996) found that children with dyscalculia had difficulties in subitizing. They appeared to use a counting strategy instead. Landerl et al. (2004) reported that those with subitizing deficits were also impaired in arithmetic tasks and in differentiating numbers. Similar results were obtained by Schleifer and Landerl (2011).

Reigosa-Crespo et al. (2012) studied 11, 652 children in Havana, Cuba in grades ranging from 2nd to 9th grade. 3.4% of these children showed deficits in basic numerical capacities, including subitizing. Almost all of these children also had difficulty in calculation. A further l 9.35% had deficits in calculation, but not in basic numerical abilities. Those with basic numerical difficulties tended to have more severe calculation difficulties than the others with poor calculation, and differed from them in some other important ways: for example, most were boys.

Estevez-Perez et al. (2019) followed up this study and also looked at typically developing children. They found that subitizing, verbal counting and numerical magnitude comparison all predicted early and later acquisition of arithmetic and were impaired in children with low arithmetic achievement and with developmental dyscalculia. They also found that children with both low arithmetic achievement and poor, subitizing, were slower at arithmetic and relied more on compensatory strategies than children with similarly low arithmetic scores, but no subitizing deficits.

If subitizing is important in the development of some or all aspects of arithmetic, one might expect that training in subitizing might improve mathematical performance (Clements, 1999; Matsumoto, 2021). Özdem and Olkun (2019) did find that 640 min of training over an eight-week period in conceptual subitizing skills improved second- and third-grade children’s overall mathematical performance both immediately after the end of the intervention and a few months later.

Subitizing itself may be componential and not a single entity. Anobile et al. (2019a) found that simultaneous and sequential subitizing did not correlate significantly with one another in primary school children (Burr et al., 2010; Anobile et al., 2013, 2019a). Neither type of subitizing predicted either children’s mental calculation or their digit magnitude knowledge. By contrast, estimation of larger numerosities did predict children’s arithmetic.

Anobile et al. (2019b) also argue against the mainstream view that there are just two visual numerosity processing systems: one for small numerosities (subitizing) and one for larger numerosities (estimation). They consider that there may in fact be *three* such systems: one for numerosities under 4; one for high numerosities, and one for intermediate numerosities such as those between 10 and 20. They suggest that the intermediate numerosity system may rely less on attention than either the low or high numerosity system. As evidence for this proposal, they use findings that performing a distracting concurrent task impaired typical adults’ numerosity judgment performance much more for low or high numerosities than for intermediate numerosities. They also studied a simultagnosic patient and found that his ability to compare either very low or very high numerosities was seriously impaired, while showing relatively preserved ability to compare intermediate numerosities.

## Non-symbolic magnitude approximation and comparison

A crucial question is whether the ability to compare non-symbolic number magnitudes (e.g., quantities of dots) is a strong predictor of numerical abilities, as might be suggested by Dehaene’s (1997) theory. We can represent and compare number sizes both with regard to displays of sets of objects such as dots (3 dots are more than 2 dots; 50 dots are more than 40 dots; etc.), and with regard to symbols such as numerals (3 is more than 2; 50 is more than 40; etc.). How much does the latter depend on the former? Some studies suggest that the ability to deal with and compare non-symbolic number magnitudes is related to mathematics achievement, but that the relationship is relatively weak, and in particular less strong than the relationship of symbolic number magnitude understanding to mathematics achievement (Gilmore et al., 2010; Fazio et al., 2014).

Lyons et al. (2012) speak of “symbolic estrangement”: a dissociation, possibly increasing with age, between symbolic and non-symbolic comparison abilities. They suggest that symbolic number representation may initially be closely based on non-symbolic number representation, but that the two abilities become increasingly independent with age. A few studies have suggested that there is only a limited relationship between the two even at an early age. Soltész et al. (2010) looked at different numerical abilities in 4-to-7-year-olds and found that non-symbolic numerical magnitude discrimination did not correlate significantly with verbal counting or arithmetic even during that age range. Coolen et al. (2022) gave children two computerized Approximate Number System tasks, two executive function tasks, a verbal skills task, two intelligence subscales, and a mathematics achievement task (involving global, formal, and informal mathematics achievement). Results demonstrated that, when controlling for intelligence and visuospatial memory, the relation between ANS acuity and mathematics achievement ceased to exist. Negen and Sarnecka (2015) argued that there is no real relationship between ANS acuity and any sort of exact number knowledge. In their view, such relationships only appear to exist because some studies have inadvertently allowed children to answer correctly based on the size rather than the number of dots in the display and because young children may not understand the phrase “more dots” to mean numerically more. In some studies where these potential problems have been controlled for, the correlation between children’s ANS acuity and their exact-number knowledge disappears.

Most studies do, however suggest a predictive role for non-symbolic number comparison with regard to later symbolic arithmetical abilities (Halberda et al., 2008; Mazzocco et al., 2011; Libertus et al., 2012; Chen and Li, 2014; Libertus et al., 2016; Xenidou-Dervou et al., 2017). In line with a multiple-component theory of arithmetic, non-symbolic quantity comparison seems to predict some components of mathematics better than others. Libertus et al. (2011) carried out a longitudinal study of the numerical abilities of children starting between the ages of 3 and 7, who were tested at four points during the following 2 years. Their ability to compare quantities of dots correlated with and predicted informal but not formal mathematical abilities. In particular, it predicted their verbal counting, their ability to compare numerical symbols, and to link arithmetic problems to concrete representations, but not their ability to retrieve number facts or to understand place value.

Several studies have shown that children with a diagnosis of dyscalculia are often impaired at approximate comparison of sets of dots (Price et al., 2007; Piazza et al., 2010). Some studies have, however, given conflicting results. For example, Rousselle & Noel found that children with a diagnosis of dyscalculia had problems in comparing numerals but not sets of dots. Iuculano et al. (2008) found that of two children with a diagnosis of dyscalculia, one had difficulties with subitizing but not approximation and one with approximation but not subitizing.

There is, indeed, increasing evidence that non-symbolic magnitude comparison may not be totally domain-specific, but may also depend on executive functions, such as inhibition, enabling one to focus on the dimension of quantity and to ignore other aspects of stimuli, such as contour and area. Indeed, numerosity-irrelevant perceptual properties of continuous quantity have been found to increase the difficulty of numerical processing not only in young children but even in adults.

Merkley and Scerif (2015) trained forty undergraduate students to associate novel abstract symbols with numerical magnitudes. The numerical arrays either involved numerosities that were congruent with surface area or incongruent with surface area. Comparisons of symbols associated with incongruent area and numerosity were the most difficult for the participants. Thus, the need to ignore irrelevant perceptual information may make demands on executive functions at all ages and levels. This brings us to the next main topic of this article: the role of domain general abilities in numerical cognition.

## What is the role of domain-general versus domain-specific abilities?

Another important question to be discussed here, with regard to the componential nature of arithmetical and numerical abilities, is whether discrepancies between different numerical tasks are most likely to reflect strengths and weaknesses in domain-specific numerical abilities or to be secondary to strengths and weaknesses in domain-general cognitive abilities. This question arises both with regard to typically developing children and adults and with regard to people with developmental and acquired dyscalculia.

## Domain-general abilities

Domain general abilities make a significant contribution to numerical and arithmetical cognition. At one time, the main domain-general ability studied with regard to numerical cognition was logical reasoning. For example, Piaget (1952) considered the development of children’s understanding of number to depend on their stage of logical development. Logical reasoning is still considered to be important to arithmetical cognition, but current research on the influence of domain general abilities on arithmetic tends to focus on attention, executive functions and working memory. In addition, there is increasing evidence that pattern recognition and pattern construction are very important to early numerical development (Rittle-Johnson et al., 2019; Wijns et al., 2021; Di Lonardo Burr et al., 2022).

Domain general abilities may help to explain discrepancies between different components of arithmetic itself: for example, between different operations. There are often alternative possible explanations for discrepancies between different operations, which relate to more domain-general abilities. Multiplication may dissociate from other operations because it is usually learned in ways that emphasize verbal rote learning to a greater extent than other operations, and thus may rely more than other operation on verbal skills. Guez et al. (2023) carried out a longitudinal study of 358 American children from preschool (5; 5 years) to age 11. They found that preschool language skills, but not visuospatial skills, predicted multiplication skills at age 11, whereas preschool visuospatial skills, but not language skills, predicted addition and subtraction skills at age 11. Carota et al. (2013) proposed that their patient’s particular difficulty with borrowing in subtraction might be due to a deficit in the executive function of inhibition.

Domain general abilities appear to play an important role, and to interact with domain specific abilities, in an even more complex way with regard to components of arithmetical cognition in a broader sense. A number of studies have indicated the importance of both domain general and domain specific skills in early mathematical development. Attentional abilities are generally found to be important to arithmetical development (LeFevre et al., 2013). Hassinger-Das et al. (2014) studied 107 children, who performed poorly on number sense tests in kindergarten (5 to 6 years) and followed them up into first grade (6 to 7 years). Both attention problems and executive function in kindergarten predicted first grade outcomes. Attention problems in kindergarten were stronger predictors than executive function tests of calculation in first grade, while executive function tests were stronger predictors than attention problems of applied problem solving in first grade.

Most studies show that *working memory* is a significant predictor of arithmetic (Raghubar et al., 2010). Both verbal and visuospatial working memory are generally found to be important predictors of arithmetic, though there are conflicting findings about which is a stronger predictor and how this may relate to age. Wilson and Swanson (2001) found that arithmetical ability showed similar levels of correlation with working memory across a wide range of age groups. Including both children and adults. At all ages, arithmetic was more closely related to verbal working memory than to visual–spatial working memory. McKenzie et al. (2003) found that 6-year-olds’ arithmetic performance was more disrupted by visual–spatial than verbal interference, interference whereas 8-year-olds’ arithmetic performance was more disrupted by verbal than visual–spatial interference. By contrast, some other studies suggest that as children grow older, the importance of visual–spatial working memory to their arithmetic increases. Henry and MacLean (2002) found that 7- and 8-year-olds’ arithmetical reasoning was best predicted by “central executive” tasks, with some added contribution from word span and digit span. On the other hand, 11- and 12-year-olds’ arithmetical reasoning was best predicted by visual memory, with some additional contribution from word span and digit span. A meta-analysis by Zhang et al. (2023) indicated that arithmetic correlated more with verbal working memory than with visual–spatial working memory. The role of verbal working memory declined with age during the primary school years, while correlations between arithmetic and aspects of spatial working memory remained stable over the age range.

Different aspects of working memory may predict different aspects of mathematical development and performance. Simmons et al. (2012) gave 90 British children a working memory battery and some tests of mathematical skills. 41 were in Year 1 (5–6 years of age) and 49 were in Year 3 (7–8 years of age). Working memory as a whole explained significant amounts of variance in number writing, magnitude judgment, and single-digit arithmetic. Different components of working memory had different relationships with the different mathematical skills. Visual–spatial sketchpad (VSSP) functioning predicted unique variance in magnitude judgments and number writing. Central executive functioning explained unique variance in the addition accuracy of Year 1 children. Phonological loop functioning came just short of significantly predicting Year 3 multiplication. The results are consistent with the VSSP having a significant role in the development of number writing and magnitude judgments, but less of a role in early arithmetic.

Allen and Dowker (2022) found that derived fact strategy use was related to written arithmetic but not to mental arithmetic, and that visuospatial working memory predicted both oral and written arithmetic but did not predict use of derived fact strategies. These results must be treated with caution as the sample was quite small; it will be necessary to replicate them with a larger sample. Nonetheless, they do suggest that predictors may differ for different components of arithmetic. The meta-analysis by Zhang et al. (2023) indicated that verbal working memory was more closely related to addition and subtraction than to multiplication and division.

As well as working memory. Other executive functions are also highly important to mathematical skills Many studies indicate that executive functions are related both cross-sectionally and longitudinally to arithmetical performance (Mazzocco and Kover, 2007; Passolunghi et al., 2007; Best et al., 2011; Clark et al., 2013; Bull and Lee, 2014). Several studies have indicated that among executive functions, inhibition and shifting appear to be stronger predictors and correlates of arithmetic than maintenance is Geary et al. (2012) and McDonald and Berg (2018). Inhibitory control seems to play a particularly important role: For example. Bull and Scerif (2001), Bull et al. (1999), and McKenzie et al. (2003) found that 6-to-8-year-olds’ performance on mathematical tasks was significantly related to the ability to attend to relevant information and ignore irrelevant information, while not correlating significantly with auditory verbal short-term memory, with visual sequential memory, or with dual task performance.

The role of inhibition might seem at first. to be particularly important for mathematical skills such as word problem solving, where it is necessary to sift text for relevant information. However, its importance is by no means confined to such verbal tasks and can even be seen in very young children’s elementary numerical abilities. Merkley et al. (2016) found a relationship between inhibition in the Animal Stroop Test and numerical abilities in pre-schoolers. Such findings are probably due to the fact that, as suggested above, even the most elementary numerical tasks require attending to those aspects of the stimulus that are relevant to number, and ignoring irrelevant cues such as area, contour, and the size of individual items.

There is also some evidence that executive functions in general, and inhibition in particular, are particularly important predictors of *specific* aspects of numerical ability., though the evidence is conflicting as regards which aspects they predict, and how these are related to one another. Fuhs et al. (2016) found that that executive function in kindergarten children predicted their arithmetic 2 years later in second grade, but mostly in an indirect way. Executive function predicted set size recognition, which in turn predicted arithmetic. By contrast, Clayton and Gilmore (2015) found that executive function predicted both quantity estimation and arithmetical skills, but that there was almost no relationship between quantity estimation and arithmetical skills. There may be age differences with regard to the components of arithmetic that executive functions predict. Gilmore et al. (2015) found that inhibition predicted mainly procedural aspects of arithmetic in younger primary school children, and mainly conceptual aspects in older children.

There is now considerable interest in the associations between mathematical performance and both domain general and domain specific abilities. Most studies have dealt with the extent to which children’s mathematical performance and progress are predicted by such abilities. Overall, studies indicate that both are important, but it is difficult to draw firm conclusions about the relative importance of different predictors. This is because studies differ with regard to the exact domain-specific abilities being investigated; the exact domain-general abilities being investigated; the mathematical performance measures being used; the children’s age group; and the culture and school curriculum in which they are operating.

Chu et al. (2016) Investigated the contributions of domain-general and domain-specific abilities to progress in mathematics from first grade (age 6 to 7) through eighth grade (age 13 to 14) Domain general measures included first grade IQ and working memory test scores and prior reading achievement Domain-specific measures included prior grade mathematics achievement and tests of prior grade number knowledge, addition skills, and fraction knowledge. Domain-general abilities as a whole were similarly important predictors of subsequent achievement in all grades; but working memory was an increasingly important predictor of achievement in later grades. Prior mathematical measures also became increasingly important to subsequent mathematics achievement in later grades. This seemed to be mainly due to fraction knowledge becoming increasingly important in later grades. The predictive importance of number knowledge and arithmetic skills did not vary with grade. In the early grades, progress in mathematics was predicted more strongly by domain-general abilities than by domain-specific abilities. In the later grades, domain- general and domain-specific abilities were equally strong predictors in the later grades. These findings could perhaps reflect the fact that the domain-specific abilities included school-taught skills and prior school mathematics achievement, whose role in subsequent mathematical achievement might be expected to increase during the school years.

Fuchs et al. (2010a,b) assessed children at the beginning of first grade (approximately 6 years old) on measures of both subitizing small numbers and approximate representation of large numbers, and also on measures of working memory (phonological loop, visual spatial sketchpad and central executive), processing speed, attentive behavior and listening comprehension. The children were tested at the end of first grade on procedural calculations and word problems. Both domain-specific and domain-general skills were significant predictors of word problem solving, while only domain specific skills were significant predictors of procedural calculation.

Spencer et al. (2022) investigated domain-general and domain-specific skills as predictors of both mathematics and reading, They assessed first-grade domain-general abilities (nonverbal reasoning, processing speed, working memory, and attentive behavior); domain-specific mathematical abilities (calculation skill, word-problem solving, and numerical cognition) and domain-specific literacy-related abilities (word-reading fluency and listening comprehension). They investigated the extent to which these predicted second-grade academic outcomes (calculations, word-problem solving, and word reading).

Path analysis mediation models indicated that all the predictors were significant, but that numerical cognition, mediated the effects of processing speed, working memory, calculation skill, word-problem solving, and attentive behavior on all three outcomes. Spencer et al. (2022) suggested that numerical cognition may not only serve as a domain-specific predictor of mathematical skills, but may also predict reading, perhaps because it reflects ease of forming symbol-concept associations.

Most studies in the area have involved British or American pupils. It is possible that different countries and cultures might show different relationships between mathematical achievement and domain specific and domain general predictors, both because of differences in the curriculum and because of differences in out-of-school experiences and activities. Chan and Ho (2010) studied Chinese children with and without mathematical difficulties in two age groups: 7 to 8, and 9 to 11. The children were tested on four domain-specific skills: arithmetic procedural skills, number fact retrieval, place value concept, and number sense, and two domain-general skills: working memory and processing speed. The children with mathematical difficulties performed worse than those without mathematical difficulties on all the skills tested. Stepwise discriminant analyzes showed that the domain specific skills of number fact retrieval and place value concept were particularly important in differentiating the children with and without mathematical difficulties. It needs to be remembered that, unlike many studies, this study involved not dimensional measures of mathematical achievement, but a binary comparison between groups with and without mathematical difficulties, and the results could be influenced by the ways in which children were assessed as having mathematical difficulties.

Studies of patients also suggest that both domain-general and domain-specific abilities are important to performance in basic numerical tasks. Roquet et al. (2020) compared numerosity estimation on a dot array comparison task in 50 patients with Alzheimer’s disease and 48 healthy older adults. The patients with Alzheimer’s disease performed significantly worse than the healthy controls on the task, especially for small-ratio comparisons. They performed worse for incongruent items (where there was a mismatch between numerosity and area) than for congruent items (where there was no such mismatch), while the controls showed no such effects. The Alzheimer’s patients’ impaired performance on the dot array comparison task correlated with their performance on a 1 to 1,000 number line task, and also with a Simon task involving inhibition of cognitive interference. Thus, numerosity estimation is impaired in patients with Alzheimer’s disease and this seems to be related to impairments in both domain-general and domain-specific abilities.

## Cognitive control may itself be multi-componential

Merkley et al. (2017) argue that cognitive control is important to early numerical development, and that cognitive control is itself multi-componential. Two particularly important aspects of cognitive control are top-down executive control of attention and bottom-up saliency-driven attention orienting. Merkley et al. (2017) argue that two interact with each other, as well as with perception and memory over the course of development. They propose that the development of the concept of number is driven in part by the interaction between the development of selective attention to non-symbolic numerosity and the acquisition of the meaning of number words in early childhood.

## Can we always distinguish sharply between domain-general and domain-specific?

The distinction between “domain-general” and “domain-specific” may itself be over-simplified, Merkley et al. (2017) point out that cognitive control operates and develops not in isolation, but in conjunction with the development of relevant domain-specific knowledge (e.g., Johnson, 2011; Amso and Scerif, 2015). Wilkey et al. (2020) have pointed out that executive functions demonstrated in a numerical context are more predictive of arithmetic than those demonstrated outside such a context.

Some researchers are now investigating the role of such “number specific executive functions” separately from executive functions in a non-numerical context, and domain-specific numerical abilities. For example. Wongupparaj and Cohen Kadosh (2022) gave 6-and 7-year-old children in Thailand, where formal schooling begins at 7, tests of domain-specific numerical abilities, number-specific executive functions and mathematical skills. In both preschool and primary school children, both domain-specific numerical abilities and number-specific executive functions predicted mathematical performance. Among domain-specific numerical abilities, number comparison and mental number line tasks were particularly important predictors. Findings of bidirectional longitudinal relationships between executive functions and numerical abilities also give support to the view that the domain-general and domain-specific cannot always be regarded as sharply distinct.

## Conclusion

Components of arithmetical cognition come into several important categories, shown in Table 1. The categories include non-symbolic quantity representation and processing; symbolic quantity representation and processing; counting procedures and principles; arithmetic operations; arithmetical knowledge and understanding; multiple forms and applications of conceptual knowledge of arithmetic; and domain-general abilities such as attention, executive functions and working memory. There is much evidence that different components can and often do show considerable functional independence, not only in developmental and acquired dyscalculia, but in typically achieving children and adults. At the same time, it is possible to find complex interactions and bidirectional relationships between the different components, including between domain-specific and apparently domain-general abilities.

**Table 1:** Important categories of components of arithmetical cognition.

Component category	Examples of components within category
Non-symbolic quantity representation and processing	Subitizing (small sets) Magnitude approximation (larger sets) Possible intermediate quantity recognition (Anobile et al., 2019a,b)
Symbolic quantity representation and processing	Quantity representation by words Quantity representation by numerals Translation between words and numerals (transcoding) Translation between words and non-symbolic quantities Translation between numerals and non-symbolic quantities
Counting procedures and principles	Verbal counting procedures Object counting procedures Use of ordinal number Cardinal word principle Order irrelevance principle
Arithmetic operations	Addition Subtraction Multiplication Division Carrying procedures in addition Carrying procedures in multiplication Borrowing procedures in subtraction Fraction and decimal arithmetic
Arithmetical knowledge and understanding	Factual knowledge Procedural knowledge (related to arithmetical operations above) Conceptual knowledge
Examples of conceptual knowledge	Arithmetical principles: e.g. commutativity; associativity; distributivity; addition-subtraction inverse principle; multiplication-division inverse principle Use of arithmetical principles in derived fact strategies Understanding the base 10 system Arithmetical estimation Comprehending and solving word problems Conceptual understanding of fractions
Domain-general abilities	Pattern recognition and construction Logical reasoning Attention Executive functions, including inhibition, shifting and maintenance Verbal working memory Visuospatial working memory Interface with domain-specific abilities: ‘Number-specific executive functions’ (Wilkey et al., 2020)

## Areas for further research

We have gained a lot of knowledge about numerical cognition and its componential nature; but there is a great deal that still needs to be discovered. In particular, we need to learn more about the origins in infancy of subitizing and approximate magnitude comparison, the extent to which these interact, the extent to which they may be further divisible, and the extent and ways in which they themselves may develop with age and the extent to which they may influence later-developing components.

There also needs to be a lot more research on exactly how domain-general and domain-specific abilities contribute to mathematical development, and how they interact with one another. It is certain that both domain-general and domain-specific abilities are involved. There are, however, many unanswered questions about how they relate to one another; the extent to which they may have differential effects on different arithmetical skills; the extent to which they may have differential effects at different ages; the extent to which they may play different roles in developmental dyscalculia and in typical development; and the relative importance of different domain-general abilities. As regards the latter, the greatest focus in research has been on working memory. Other executive functions, attention, and spatial ability, but, as Agostini et al. (2022) point out, there is a need for further research on other cognitive functions, for example including long-term memory and phonological awareness (Jordan et al., 2010). There also needs to be more research on possible bidirectional relationships between arithmetical learning and both domain-specific and domain-general abilities, given the increasing evidence for a bidirectional relationship between the development of executive functions and numerical abilities in children (Welsh et al., 2010; Fuhs et al., 2016; Wilkey et al., 2020; Coolen et al., 2021). A greater understanding of these issues could make a significant contribution to the development of educational interventions.

It is also important to study numerical development and cognition in a wider variety of environments, contexts and cultures (Santos et al., 2022).

Finally, although this article deals just with numerical cognition, it is important to study other aspects of mathematical cognition, including geometry, spatial cognition, pattern awareness and algebra-related concepts. Most of these have received far less study than number. It is also important to gain a greater understanding of relationships between emotional and cognitive factors in mathematics (Cipora et al., 2022).

## Author contributions

The author confirms being the sole contributor of this work and has approved it for publication.

## Conflict of interest

The author declares that the research was conducted in the absence of any commercial or financial relationships that could be construed as a potential conflict of interest.

## Publisher’s note

All claims expressed in this article are solely those of the authors and do not necessarily represent those of their affiliated organizations, or those of the publisher, the editors and the reviewers. Any product that may be evaluated in this article, or claim that may be made by its manufacturer, is not guaranteed or endorsed by the publisher.
